# Supplementary material for: The Recovery of Soybean Plants after Short-Term Cadmium Stress
Source: Plants (Basel). 2020 Jun 22;9(6):782. doi: 10.3390/plants9060782 (PMC7356936; doi:10.3390/plants9060782)
Supplement: Supplementary file 1 [file plants-09-00782-s001.pdf]

Table S1. Percentage of Cd content in particular organs of recovered plants. TF – root-to-shoot translocation factor. Statistically significant differences in Cd accumulation in specific organs by p = 0.05 are marked with different letters.

	**Seedlings**	
	**Treated with 10 mg/L**	**Treated with 25 mg/L**
roots	81% ± 6 [a]	93% ±2 [a]
hypocotyls	19% ± 6 [b]	7% ± 2 [b]
TF	0.3 ± 0.1	0.1 ± 0.02
	**Recovered plants**	
	**Treated with 10 mg/L**	**Treated with 25 mg/L**
roots	62% ± 7 [a]	85% ± 4 [a]
stems	33% ± 4 [b]	11% ± 3 [b]
fist leaves	5% ± 1 [c]	4% ± 1 [c]
TF	0.7±0.21	0.4 ±0.19

Table S2. Content of non-essential elements in the roots, stems and leaves of soybean plants after the recovery period from Cd stress. Values are means of 2–3 repetitions ± SE. No significant differences between Cd-treated and control plants were noted.

**Element**	**Concentration (µg/g DW)**		
	**Control**	**Cd 10 mg/L**	**Cd 25 mg/L**
		**Roots**	
**Aluminum (Al)**	**102** ± 6	**113** ± 8	**94** ± 2
**Arsenic (As)**	**2** ± 0	**1.8** ± 0	**2** ± 0
**Bromine (Br)**	**9** ± 0	**8.3** ± 1	**9** ± 1
**Rubidium (Rb)**	**21** ± 1	**22** ± 2	**18** ± 0
		**Stems**	
**Aluminum (Al)**	**56** ± 3	**51** ± 2	**41** ± 4
**Arsenic (As)**	**0.5** ± 0	**0.4** ± 0	**0.5** ± 0.2
**Bromine (Br)**	**8** ± 3	**9** ± 1	**9** ± 1
**Rubidium (Rb)**	**10** ± 1	**12** ± 1	**14** ± 6
		**Leaves**	
**Aluminum (Al)**	**69** ± 11	**72** ± 6	**50** ± 3
**Arsenic (As)**	**0.3** ± 0	**0.4** ± 0	**1** ± 1
**Bromine (Br)**	**4** ± 1	**1.5** ± 0	**4** ± 1
**Rubidium (Rb)**	**12** ± 0	**13** ± 1	**14** ± 0
